# Implementing Performance Accommodation Mechanisms in Online BCI for Stroke Rehabilitation: A Study on Perceived Control and Frustration

**DOI:** 10.3390/s22239051

**Published:** 2022-11-22

**Authors:** Mads Jochumsen, Bastian Ilsø Hougaard, Mathias Sand Kristensen, Hendrik Knoche

**Affiliations:** 1 Department of Health Science and Technology, Aalborg University, 9000 Aalborg, Denmark; 2 Department of Architecture, Design and Media Technology, Aalborg University, 9000 Aalborg, Denmark

**Keywords:** brain–computer interface, motor imagery, gamification, stroke rehabilitation, frustration, perceived control, performance accommodation mechanisms, game design

## Abstract

Brain–computer interfaces (BCIs) are successfully used for stroke rehabilitation, but the training is repetitive and patients can lose the motivation to train. Moreover, controlling the BCI may be difficult, which causes frustration and leads to even worse control. Patients might not adhere to the regimen due to frustration and lack of motivation/engagement. The aim of this study was to implement three performance accommodation mechanisms (PAMs) in an online motor imagery-based BCI to aid people and evaluate their perceived control and frustration. Nineteen healthy participants controlled a fishing game with a BCI in four conditions: (1) no help, (2) augmented success (augmented successful BCI-attempt), (3) mitigated failure (turn unsuccessful BCI-attempt into neutral output), and (4) override input (turn unsuccessful BCI-attempt into successful output). Each condition was followed-up and assessed with Likert-scale questionnaires and a post-experiment interview. Perceived control and frustration were best predicted by the amount of positive feedback the participant received. PAM-help increased perceived control for poor BCI-users but decreased it for good BCI-users. The input override PAM frustrated the users the most, and they differed in how they wanted to be helped. By using PAMs, developers have more freedom to create engaging stroke rehabilitation games.

## 1. Introduction

A stroke is globally one of the leading causes of acquired disability among adults [1]. However, the heterogeneity of the injury complicates finding a single treatment that is effective for all patients and the effects of existing treatment options are limited [2]. However, in recent years, several new rehabilitation techniques have been proposed, which rely on the induction of plasticity and motor learning principles [3,4,5]. One proposed technique that has shown promising results is the brain–computer interface (BCI) [6,7,8]. It was shown in many studies that BCIs can be used for inducing Hebbian-associated plasticity by triggering electrical stimulation [9,10,11,12], rehabilitation robots [13,14], or exoskeletons [15] based on movement-related cortical activities through either motor imagery (MI) or attempted movements [16]. Improvements in functional scores such as the Fugl-Meyer Score have consistently been reported for upper and lower limbs (see, e.g., [17,18] for recent reviews). BCI training can be effective, but as for many other rehabilitation techniques, repetitive training is needed, and the outcome is likely to be correlated with the amount of performed training. The repetitive training may cause boredom in the patients, which eventually can lead to patients not adhering to the regimen [19]. A potential solution to keep the patients engaged and motivated to maintain the training efforts can be through gamification [20], which was used successfully in various other rehabilitation scenarios [21,22]. To introduce gamification in BCI-based rehabilitation, patients need to be able to provide input to activate the lesioned brain area to maximize the effect of the rehabilitation [7]. However, 10–30% of all individuals cannot operate a BCI satisfactorily for control and communication purposes, i.e., when achieving recognition rates less than 70% [23]. It should be noted though that lower recognition rates still induce plasticity [8], although a better BCI performance was suggested to improve the induction of plasticity [9]. The BCI performance may be enhanced in various ways by selecting the optimal pre-processing techniques [24,25,26], features [25,27,28,29,30], classifiers [29,31], or by focusing on user instructions and training [23,32,33]. By improving the BCI performance, the patients’ perceived control and frustration improve as well [34,35,36,37,38,39,40], which may help them maintain interest in the training. Moreover, frustration has a detrimental effect on BCI performance with increasing frustration leading to worse BCI performance [39]. Despite the use of optimal signal processing techniques or learning principles, the BCI performance may still be poor for some users, or other factors may impede the BCI performance, such as incompetence or fear [41]. A way to tackle this is by injecting concealed, artificial, positive feedback and in this way improve the perceived BCI performance [34,35]. This approach can only be implemented in a meaningful way in synchronous BCIs with binary input (MI vs. idle activity) [35]. Alternatively, game mechanics can be used to assist users, such that they maintain interest in the training, and the mechanics conceal the actual BCI performance. The game mechanics represent a type of dynamic difficulty adjustment [42], which regulate the game’s challenge to accommodate for imperfect user input, and are named performance accommodation mechanisms (PAMs) [43]. PAMs are used to match the challenge of the game to the player’s skill level. If the game’s challenge is sufficiently but not too high, players can enter a flow state in which they feel challenged but will likely succeed in making the interaction engaging [44]. This could be important in a BCI training context where there is great variability in the BCI skill levels. Flow was reported to account for a major part of the enjoyment of playing games [45,46].

A PAM may be defined in the following way: “*A game mechanism to increase the player’s enjoyment by lowering the game’s challenge level to accommodate for poor performance of the player, input device or system*” [43]. PAMs may be divided into five overall groups (although other smaller and more specific groupings may exist): Augmented success, mitigated failure, input override, rule change, and shared control [43]. In this study, we focus on the first three listed PAMs. Augmented success provides the user with an outcome that is better than what normally can be expected from a successful input, e.g., this mechanism was implemented as power-ups or boosts in driving games. Mitigated failures transform failed inputs to outputs that are between failure and success, such that failed inputs are not penalized but not successful either. An example of this mechanism in a shooting game could be that a low-performing player is not losing as much health as if the mechanism was not activated. Input override can replace a failed input with a system-generated successful input, e.g., this mechanism can be used in a targeting shooting task where failed inputs still lead to an instant lock on the nearest target. These PAMs could be used to create engaging games that allow patients with poor BCI control to experience more enjoyable rehabilitation training sessions. However, it is unknown how these PAMs affect perceived control and frustration in a BCI context. To that end, this study implements PAMs in an MI-controlled online BCI game and investigates how each PAM affects the levels of perceived control and frustration as well as exploring the qualitative aspects of using such PAMs.

## 2. Materials and Methods

### 2.1. Participants

A total of 19 healthy participants participated in this study (7 women and 12 men with a mean age of 27±8 years). All participants provided their informed consent prior to participation. Prior to the experiment, the participants were instructed on how to perform kinesthetic or first-person MI [16].

### 2.2. Brain–Computer Interface

**Figure 1:**
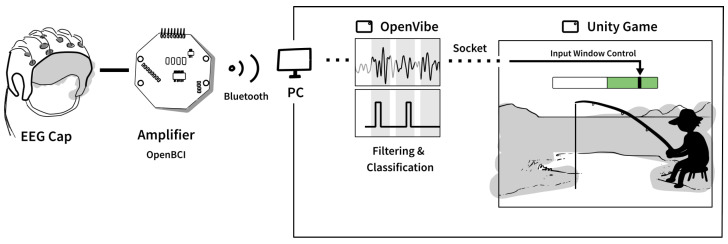
Data flow from the BCI cap to the fishing game developed in Unity. The BCI only controls the game when the black cursor is within the input window, marked by the green area on a bar displayed in the fishing game.

The BCI in this study was based on kinesthetic MI of a palmar grasp of the right hand and implemented using the “Motor Imagery BCI” scenario in OpenViBE [47]. A similar setup was used previously (see, e.g., [15,35,48]). Continuous EEG was recorded using a cap with sintered Ag/AgCl electrodes (OpenBCI, USA) and amplified using a Cyton Biosensing Board (OpenBCI, USA). The EEG was recorded from F3, F4, C3, Cz, C4, P3, and P4 according to the International 10–20 System. The electrodes were grounded at CPz and referenced to AFz. The EEG was sampled at 250 Hz. The amplified EEG was transmitted through Bluetooth to a computer running the OpenViBE software. The EEG was bandpass filtered between 8 and 30 Hz with a 5th-order Butterworth filter to reduce the electrical activity outside the mu (8–12 Hz) and beta (13–30) frequency ranges for enhancing the event-related desynchronization [49]. This was followed up with a common spatial pattern filter that was applied to maximize the difference in spectral power between the two classes (MI vs. idle activity). The bandpower was obtained from the CSP-filtered data from each electrode and used as input for a linear discriminant analysis classifier. The filter coefficients for the CSP filter and the parameters for the decision boundary were extracted from calibration data. The linear discriminant analysis classifier was trained using five-fold cross-validation. Every 1/16 s the BCI system calculated a value between 0 and 1, and if the value exceeded a subject-specific threshold of 0.5 s it was considered as MI. The subject-specific threshold was determined based on the threshold leading to the highest offline classification threshold. This threshold was used in a short online test of the BCI system (<5 min) before the actual testing began to adjust it if necessary to obtain a trade-off between the number of true positive and false positive detections. When MI of a palmar grasp was detected in the experimental sessions a trigger was sent to unity through a TCP socket in OpenViBE. Figure 1 visualizes the complete communication relationship between the BCI and the game.

### 2.3. Game

**Figure 2:**
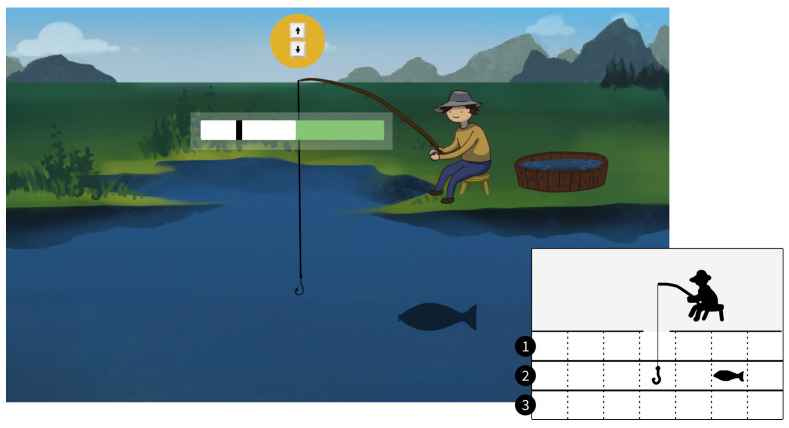
In the fishing game, participants control a fisherman reeling fish. Participants use arrow keys to move the hook up and down between three lanes. A fish may appear in a random lane from either left or right side and may swim into the participant’s hook. The BCI input window then begins and the participant may then perform MI when the black cursor is within the green area.

The participants in this study played a game where three implementations of the different PAMs/help could be integrated. The participants played a custom-made fishing game where they controlled a fisherman and had to catch as many fish as possible from a lake. The player had to move the hook up and down using the up and down keys on a keyboard, to catch the fish, which swam at three different depths in the lake (visualized in Figure 2. When the fish swam into the hook, it was hooked and a progress bar was shown. Then the player had to reel in the fish using kinesthetic MI of a palmar grasp of the right hand. To avoid conflicting movement-related brain activity associated with pressing the keys on the keyboard and reeling in the fish with MI, the MI was initiated two seconds after the last press on the keyboard. Initially, a preparation phase of two seconds was given (marked with white) followed by a two-second input window where the user had to perform MI (marked with green). A black cursor moving from left to right indicated the timing of the two phases. The input window closed when MI was detected or after two seconds if no MI was detected. When the input was closed the participants received feedback in the form of (A) the fish being reeled in (success), (B) the fish unreeling (failure), or (C) PAM activation (special). It required one to three reels to catch the fish depending on the fish’s depth in the lake. It required three unreels for the fish to escape.

#### 2.3.1. Performance Accommodation Mechanisms

The experiment evaluated three PAMs: augmented success, mitigated failure, and input override. The PAMs were implemented in the fishing game as ways to help the player reel in the fish. In the augmented success PAM condition, the fisherman eats a herb to make him stronger, which helps the player reel in the fish faster—moving up two lanes instead of one. Augmented success provides extra positive feedback, equivalent to two successful reels. In the *mitigated failure* PAM condition, the fisherman adds a clamp to the fishing rod such that the fish is prevented from escaping. At the end of a mitigated failure trial, the fish maintains the same position which can be considered neutral feedback. In this way, the fish is not caught, but it does not escape either. In the input override PAM condition, an external computer-controlled avatar in the form of a person comes in and takes over the fishing rod to reel up the fish on behalf of the fisherman. The input override provides positive feedback equivalent to a regular single successful reel. We contrasted all of these PAM conditions with a reference condition labeled as ‘normal’ in which players only received regular positive and negative feedback based on their input. Table 1 provides a full overview of the possible outcomes within each condition.

#### 2.3.2. Urn Model

**Figure 3:**
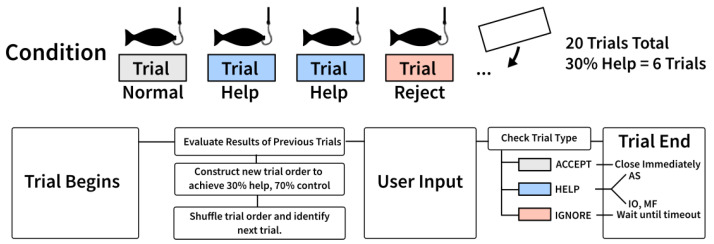
Each condition consisted of 20 trials. In the helped conditions, help trials with predefined outcomes (blue) were shuffled with normal (no PAM) trials (gray) to provide users with 30% help. Forced rejections (red) were inserted when people were succeeding above the 70% target control rate.

Each condition consisted of 20 trials in which players could attempt to reel in fish by performing MI. In the reference condition, all trials were controlled by the players’ BCI. In PAM conditions, normal trials were shuffled with 30% special trials as visualized in Figure 3. In addition, participants’ trials were rejected if they exceeded 70% control in the helped condition, to ensure all participants had similar experiences including both positive and negative feedback. To ensure that participants experienced the target rates, trials had predefined behaviors, which determined how the trial could end, visualized in the bottom flow chart in Figure 3. Rejection trials could override successful attempts if more negative feedback was needed. Special trials could override both successful and rejected attempts, except for augmented success, which required successful input from the user to augment. The order of special trials, normal trials, and rejected trials was determined by an urn model. The urn model continuously counted how many successful, failed and special trials players had and evaluate the order of upcoming trials. If the urn model decided that a player was to receive augmented success in a trial, this would require them to produce the success. If the player failed to perform MI, the urn model would evaluate the order of upcoming trials again and place an augmented success in a later trial. Trials designated for input override and mitigated failure disregarded users’ input and provided help at the end of the input window instead. This behavior was used for experimental purposes to ensure enough PAM trials were provided; in real scenarios, input override and mitigated failure only trigger when players fail to perform MI.

### 2.4. Experimental Setup

**Figure 4:**
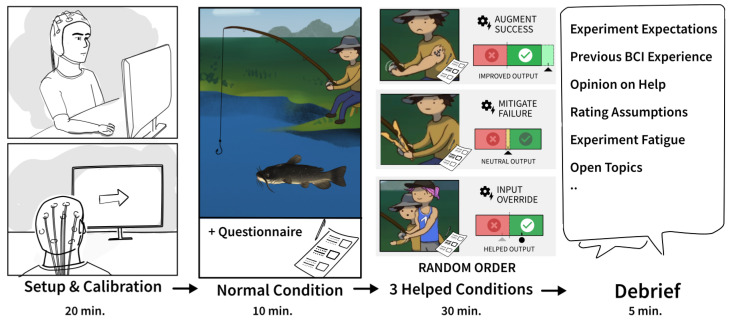
Each participant in the experiment (1) underwent BCI setup and BCI calibration, (2) played a fishing game in four conditions, starting with the normal condition, followed by (3) three helped conditions in a shuffled order. Participants were then debriefed about their experiences.

Initially, the cap was mounted on the participants and the signal quality was checked to make sure there was good signal quality (see Figure 4). In the calibration session, the participants were asked to perform MI 30 times. They were instructed to perform kinesthetic or first-person MI by recalling the sensation of doing a palmar grasp of the right hand. They were asked to maintain the imaginary contraction for four seconds while avoiding blinking or making contractions of facial muscles or other muscles. A visual cue of a red arrow pointing to the right was shown to the participants for four seconds to indicate when to start and stop the imaginary contraction. Thirty trials of idle activity were also recorded when the participants were resting, a visual cue with the text “Rest” was displayed to the participants for four seconds. Each MI trial was followed by an idle activity trial. After the BCI was calibrated the experiment started. The experiment followed a within-subject design, where participants played four conditions each (a control condition without PAM, and one condition per PAM). To avoid any order bias, we used a Latin square design for PAM conditions. The participants were introduced to one condition at a time. Prior to each condition, the facilitator introduced the condition by explaining the PAM.

- Control condition: The facilitator explained the core game. This condition was always the first condition the participants went through.
- Augmented Success: “*In this condition, the fisherman will occasionally become stronger.*”
- Mitigated Failure: “*In this condition, occasionally a clip on the fishing rod will prevent the fish from escaping.*”
- Input Override: “*In this condition, a girl will occasionally come to help you.*”

In each condition, the participants played for 20 trials and tried to catch as many fish as possible. For the final fish in each condition, if the participants had no more trials left, the fish would escape.

In accordance with previous BCI-related studies, we focused on the user experience [34,35,36,37], and the dependent variables we measured were frustration and perceived control. After each condition, participants rated on a Likert scale their perceived control (“*I felt I was in control of the fisherman reeling in the fish.*”) 1 (Strongly disagree) to 7 (Strongly agree) and frustration (“*How much frustration did you feel in this condition?*”) from 1 (Strongly absent) to 7 (Strongly pronounced). They were informed to do this while considering the condition as a whole (“*Please rate your experience as a whole during this play-through.*”). The participants were kept unaware of their actual BCI performances from their calibration and test sessions so that they would not influence their ratings.

At the end of the experiment, the participants were debriefed. First, participants were inquired as to their prior expectations of the experiment, for instance, whether they thought they would do better or worse, and how it was to control the BCI. Participants elaborated on any previous experience with BCI, to allow for grouping and rating difference checks in the analysis. Participants pointed out the hardest and easiest condition, and what their thoughts were on the PAMs. We went through their Likert scale ratings with them, to check for potential misunderstandings, i.e., prompting them to explain extreme values, which were used in the qualitative analysis to reason about outlier data points.

### 2.5. Data Analysis

#### 2.5.1. Variables

The study collected continuous data and MI detections from the BCI and event data from the game (e.g., user input and game activity). An overview of the variable pool can be found in Table 2. Each participant contributed perceived control and frustration Likert scale item scores for each of the four conditions, which was merged with the game data and analyzed in R studio. Individual conditions were reviewed to identify potential abnormalities. From the combined dataset, we selected eight variables (MI conversion rate, PAM rate, condition, positive feedback, fish caught, fish lost, fish reel, and fish unreel) to evaluate people’s ratings of perceived control and frustration. Fish unreel, fish reel, fish lost, and fish caught were included in the analysis because they represent the types of positive and negative feedback presented in the game. PAM Rate was included to analyze the impact of introducing help. In addition, we included the condition variable to analyze for differences between three types of help (augmented success, mitigated failure, and input override) and the normal condition. MI conversion rate was included to compare how users’ ability to perform MI to obtain successful trials affected perceived control and frustration ratings.

**Table 1:** Trials were manipulated by the urn model to target 30% help and limit control in help conditions. The table shows the mean % of how help conditions changed the outcomes as described in Section 2.3.1, compared to the normal condition (reference condition).

Augmented Success (AS)		Input Override (IO)		Mitigated Failure (MF)		Normal Condition	
Negative (No Change)	46%	Negative (No Change)	33%	Negative (No Change)	30%	Negative (No Change)	42%
Positive (No Change)	28%	Negative to Positive (IO)	15%	Negative to Neutral (MF)	17%	Positive (No Change)	57%
Positive to Extra Positive (AS)	14%	Positive (No Change)	37%	Positive (No Change)	40%		
Positive to Negative	12%	Positive to Positive (IO)	15%	Positive to Neutral (MF)	13%		

#### 2.5.2. Analysis Method

Many of the explanatory variables represent different ways to consider positive feedback and it is not clear which variables are better at explaining how people rate perceived control and frustration. To investigate this question, we constructed models from the variables and tested whether models, which included a variable, were significantly different to a null model without the variable present. We used cumulative link mixed models from the ordinal package [50] fitted with Laplace approximation, also known as an ordered response mixed model. We used cumulative link mixed models in our analysis because they provide a regression framework that treats observations made in the experiment’s response variables frustration and perceived control correctly as ordinal data. To counter potential pseudoreplication [51] from our repeated measures design, we used *Participant* as the basis for the null model and modeled it as random intercepts to account for by-subject baseline rating differences. We determined the most suitable model from our variables by using forward step-wise selection, which added variables based on the Akaike information criterion (AIC). We tested for significant predictors of frustration and perceived control, using Likelihood ratio tests with a *p*-value threshold of 0.05. The variables were tested as fixed effects and determined based on their known relationship in affecting control or positive feedback in the experiment.

Participant Likert scores of perceived control and frustration were summarized visually through to aid exploratory analysis. In contrast to the cumulative link mixed models, participants’ Likert scores were normalized from 1–7 to 0–1, treated numerically in tables, and visualized with linear regression for exploratory analysis.

Qualitative data included participant video recordings, game recordings, and notes taken during debriefing interviews, which we thematically analyzed for repeated patterns [52]. Due to a mistake in the experimental procedure, Participant 2 had missing data and was, therefore, excluded from the analysis.

## 3. Results

Eighteen participants played and scored four conditions, shown in Table 3. In three conditions, an urn model manipulated their experience, as summarized in Table 1.

### 3.1. Perceived Control

**Figure 5:**
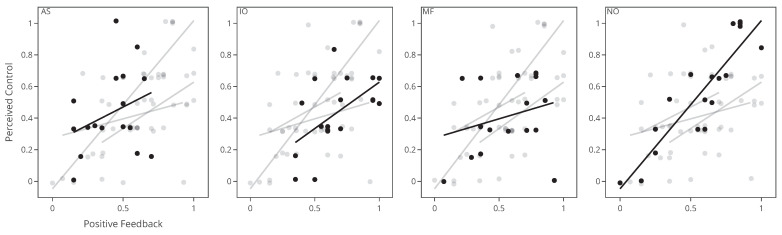

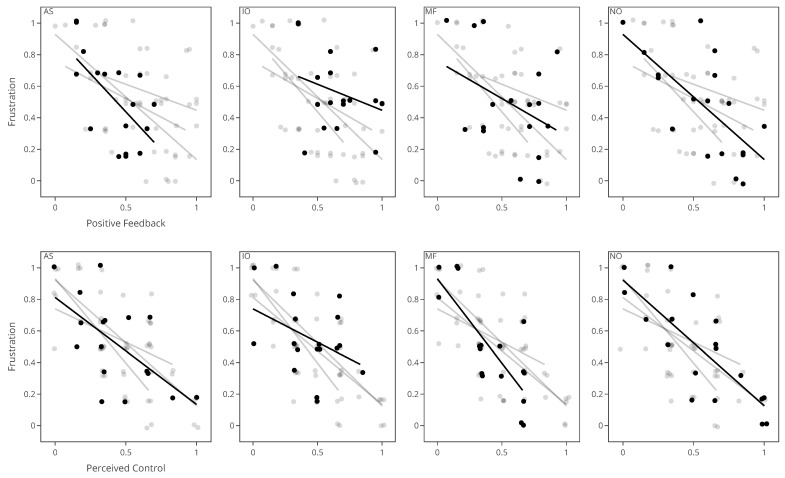
The relationship between perceived control and positive feedback is shown in the top row of each of the four conditions, while the relationship between frustration and positive feedback is shown in the middle row. In the bottom row, the relationship between frustration and perceived control is shown. AS: augmented success, IO: input override, MF: mitigated failure, and NO: normal condition without PAM help. Each data point represents the rating of a single participant.

Forward stepwise selection constructed nine significant models for perceived control, listed at the top of Table 4. Six of eight explanatory variables resulted in significant models, where *Fish Lost* performed best in terms of AIC, ML, and LR when compared to the null model. *Fish Lost* was, therefore, chosen as the null model, and to form the basis for the model construction in the forward stepwise selection, to see if the variable could be combined with others. Three of the eight fixed effects (*Fish Caught*, *Condition*, and *PAM Rate*) made significant improvements to the model with *Fish Lost*. Examination of the *Fish Lost* + *PAM Rate* model resulted in the model outcomes shown at the bottom of Table 4. Contrary to expectations, *PAM Rate* was estimated to negatively affect participants’ rating of perceived control (estimate = −7.86, *p* < 0.001)—when people received more help, their ratings generally were lower. The examination of the second-best model *Fish Lost + Condition* estimated that the negative effect came from the conditions *input override* and (estimate = −2.04, *p* = 0.004) *mitigated failure* (estimate = −2.08, *p* = 0.004), while *augmented success*’s estimate was marginally positive it did not significantly affect perceived control (Estimate = 0.2, *p* = 0.786). The negative effects of input override and mitigated failure are also evident in the top row of Figure 5, which visualizes the relationship between positive feedback and perceived control in each condition. From the visual inspection, we observed that when participants experienced more than 50% of positive feedback, they tended to favor conditions without help. Only in cases where positive feedback was low (less than 50%), did participants rate help higher in the augmented success condition.

### 3.2. Frustration

Forward stepwise selection constructed four significant models, using four of the eight explanatory variables to predict frustration, listed at the top of Table 5. Escaping fish frustrated the participants (*Fish Lost*, Estimate = 0.62, *p* = 0.003), and conversely, participants were less frustrated when they caught more fish (*fish caught*, estimate = −0.39, *p* < 0.001). However, participants’ frustration ratings were not affected by the type of help they received. For frustration, no models that included *PAM Rate* or *Condition* were different from the null model. Visual inspections of the middle row plots in Figure 5 show a clear downward relationship between frustration ratings and positive feedback for all conditions. Augmented success and normal conditions showed similar relationships while the input override showed overall higher frustration ratings despite participants receiving more positive feedback than any other condition on average (M = 0.67, SD = 0.22). Input override and mitigated failure both showed less decreasing changes in the frustration ratings as positive feedback increased, indicating that higher control did not make as much of a difference in people’s frustrations. When plotting frustration and perceived control were against each other (Figure 5, bottom row), a clear correlation was shown in all conditions with the exception of input override.

The MI conversion rate was a significant fixed effect in models of perceived control and frustration, but variables relating to in-game feedback (fish lost, fish caught) resulted in models with lower AIC, and lower ML (shown in Table 4 and Table 5). Participants had widely different MI conversion rates between 5–100% (M = 0.54, SD = 0.28).

### 3.3. Qualitative Results

Playing the control condition, several participants (10/19) found it easy to control, while a few participants (3/19) said they were learning the game in this condition, which reduced the frustration of a few participants (2/19). The in-game character taking over the fishing rod in the input override condition was frustrating for most participants (13/19), because they wanted to solve the task themselves: “*I did not want any help from the girl.*” (P7, 11, 17). Input override removed their agency “*it doesn’t really feel like my attempt when someone else was helping.*” (P2), and reduced the legitimacy of the reward “*it was less rewarding [to catch the fish] because I got help from the girl.*” (P14, 16). The mitigated failure condition highlighted participants’ failures, as they had another try but frustrated only very few (3/19). However, few participants (4/19) found the extra try less frustrating, “*the clip [mitigated failure] was encouraging because you got a second try.*” (P2). Some participants (6/19) found augmented success easy to control, as one participant mentioned that the condition felt less patronizing than the rest. Catching a fish made some participants (7/19) feel in control of the fisherman reeling in the fish. Not being able to decide when to trigger the PAMs in the three conditions caused confusion for some participants (5/19). Not being able to trigger the last action causing the fish to escape frustrated a few participants (4/19). Losing control caused a few participants (2/19) to feel frustration. P11 felt they had no control despite having good calibration.

## 4. Discussion

In this study, three PAMs (augmented success, mitigated failure, and input override) were implemented in an online MI-BCI to evaluate their effects on perceived control and frustration. The help from PAMs was perceived differently, but generally, input override frustrated participants the most since they wanted to perform the tasks by themselves, or they blamed themselves for not succeeding since they knew they were unable to trigger the BCI when they received help despite its positive outcome. Moreover, in the mitigated failure condition, a similar tendency in frustration ratings was seen since the participants were aware when they were unable to trigger the BCI, although neutral feedback was provided and that could have caused the participants to blame themselves. Both PAMs reduced the participants perceived control. The augmented success did not increase frustration or reduce perceived control. It should be noted that the participants were explicitly informed about the PAMs before they tried the conditions, so it is possible that the PAMs could be perceived differently by naive players.

Participants with lower BCI control generally rated their perceived control higher in the PAM conditions with respect to the normal condition without PAM and vice versa for participants with better BCI control. The lower ratings of perceived control for the participants with better BCI performance could be partly explained by the fact that their BCI performance could be slightly impeded in the PAM conditions. However, the participants were kept unaware of their actual BCI performance, so they could not be sure about the potential reduction of their BCI performance in the PAM conditions. They only had their own experience to judge from. Perceived control negatively correlated with frustration in all conditions, but with a weaker correlation for the input override PAM, which frustrated the participants the most. The negative correlation between perceived control and frustration is in agreement with our previous findings [34,35]. The findings regarding positive feedback as a predictor of perceived control and frustration agree with a similar study using online MI-BCI methodology with fabricated input [35]. Surrogate BCI studies have also reported that higher levels of positive feedback increase perceived control and reduce frustration [34,36,37]. Perceived control was rated differently in the PAM conditions for participants with the lowest and highest BCI performance. A similar finding was reported for BCI control with biased feedback, where users with poor BCI performances benefited from biased feedback and users with good BCI performances were impeded by this [53]. It should be noted that in the current study the BCI performance was fairly low with few participants achieving BCI performances higher than 80%. Thus, the entire spectrum of the BCI performance has not been covered sufficiently and, hence, it is unknown if similar ratings of perceived control in PAM conditions are applicable for BCI performances higher than 80%. The negative correlation between perceived control and frustration in all conditions was expected since it was shown that perceived control and frustration are inversely correlated in both able-bodied users and people with a stroke [34,35]. However, in the input override condition, a weaker negative correlation was found. This could be due to the fact that explicit help overruled the actual control and, hence, reduced the perceived control and increased frustration, which was also indicated by several participants in the qualitative analysis. Input override is similar to positive fabricated input, which has previously been shown to lead to a correlation between perceived control and frustration [35], but the difference in the current study is that input override is not concealed in the game, and the participants were informed about the input override PAM prior to the condition. Thus, it should be considered if input override should be concealed instead of being explicitly articulated in the interaction, which may reduce the frustration.

### 4.1. Methodological Considerations

As outlined, the BCI performance in this study was modest and did not cover the higher end of the spectrum. This does not necessarily mean that the participants were poor BCI performers but the design of the interaction with only a two-second input window might have been too challenging. The participants only had two seconds to produce MI, contrary to our previous study, which allowed for MI during a five-second window, which yielded a better BCI performance with recognition rates exceeding 90%. The BCI setup, hardware, and processing were identical to our previous study [35]. In hindsight, two seconds may be too little time to perform MI (or to perform more than one attempt during an input window), especially when the participants had to produce MI exceeding a specific threshold for 0.5 consecutive seconds. In future studies, we would recommend increasing the duration of the input window up to five seconds. This would increase the likelihood of a false positive detection being counted as a true positive since a longer input window means that more false positive detections can occur [12]. This risk, however, could be reduced by setting a higher threshold that has to be exceeded for a given period of time. The threshold should be set such that the number of false positive detections is minimized but that it is still possible for the user to activate the BCI. In some applications/interactions, it could be desirable to set the threshold such that either more or fewer true and false positives are accepted. In applications requiring higher thresholds, i.e., a lower number of true and false positives, PAMs may be more useful since there is more room to help the user on the contrary to applications with lower thresholds, where a higher number of true and false positives lead to many successful trials potentially making the PAMs redundant. Lastly, the BCI performance can be enhanced using other signal processing and classification methods or training the user in performing MI.

In the current study, healthy users participated, but the intended use of a gamified MI-BCI system is for stroke rehabilitation. The findings in the current study cannot be directly transferred to a population of stroke patients, which, besides motor impairments, may have cognitive impairments and different levels of technological prerequisites. Stroke patients are generally above 60 years of age, while the participants in the current study consisted of able-bodied primarily in their twenties.

### 4.2. Implications

In this study, we showed that it was possible to integrate different PAMs in a BCI paradigm that are usable and meaningful for stroke rehabilitation. The PAMs created different reactions from the users, which could be useful for designing engaging games. The findings though suggest that the use of explicit input overrides should be considered carefully to avoid frustration, but it may still be useful to use it to create engaging interactions for the user and stroke patients may perceive help differently than the able-bodied participants in this study. Augmented success can be used to highlight the successes of the users, which could strengthen motivation. By using multiple PAMs, different types of games with various designs can be created, which could support the rehabilitation efforts to get the patient to train more.

Moreover, PAMs could potentially be used in training sessions to learn to perform MI. For this application though, it is expected that augmented success and mitigated failure would be the best choices since input override will provide inaccurate feedback to the user while augmented success could reinforce the learning and mitigated failure would not discourage users.

### 4.3. Future Perspectives

In future studies where the entire performance spectra need to be covered in systematic ways, researchers could consider using surrogate BCIs that share the same characteristics as an online MI-BCI but with other more reliable input methods, such as a concealed eye-tracker (an EEG cap can be mounted and it can be conveyed to the users that blink, as picked up by the BCI) [34,35]. In this way, there is access to the ground truth and performance can be artificially controlled, such that users experience different levels of control that can be similar across the study population. As outlined previously, stroke patients differ from the participants included in the current study, and it is important to learn how stroke patients react to different PAMs, so they can be used in the best way for engaging interactions in rehabilitation. Another aspect that should be tested, is how users react to PAMs when they attend multiple training sessions. It is expected that the BCI performance could improve as a result of training and familiarization with the BCI system and interaction. In the current work, PAMs were rated differently for better-performing users compared to users with lower BCI performance. Lastly, the type of interaction should be considered if the feedback should be realistic, e.g., using a humanoid hand or if the feedback can be more abstract [48]. The former is shown to improve the ownership and perceived control over more abstract feedback, but the latter could result in more engaging or fun interactions by providing the designers of rehabilitation games with more artistic freedom.

## 5. Conclusions

This study showed that PAMs could be integrated into an online BCI based on MI, and the different PAMs could assist the participants. The amount of combined positive feedback received from regular and PAM-enhanced inputs could explain the perceived control and frustration of participants. The different PAMs can be used in a more varied and richer way to aid users with poor BCI performance beyond adding simple extra positive sham feedback. The condition that explicitly depicted input override frustrated participants the most, but it is clear that people have different preferences in how they can be helped. Within the different types of PAMs, game developers can exercise tremendous artistic freedom to create engaging interactions for BCI training that either directly manipulate the outcomes of a single action or its effect in a bigger task context.

## Figures and Tables

**Table 2:** Descriptions of dependent (response) and independent (explanatory) variables used in the analysis and their minimum (Min) and maximum values (Max), means, and standard deviation(s) (SD).

Variables	Min	Max	Mean	SD	Description
Response					
Perceived Control	0	1	0.46	0.27	Normalized 7-point Likert scale rating by participants after playing a condition.
Frustration	0	1	0.50	0.29	Normalized 7-point Likert scale rating by participants after playing a condition.
Explanatory					
MI Conv. Rate	0	1	0.54	0.28	Normalized count of trials that were caused by successful motor imagery activations in a condition.
Pos. Feedback	0	1	0.52	0.24	Normalized count of how many trials delivered a positive outcome (reeling fish, catching fish, receiving help) in a condition, regardless of cause.
Fish Caught	0	8	3.59	2.39	Count of how many fish were reeled all the way up and caught in a given condition.
Fish Lost	0	6	1.69	1.69	Count of how many fish participants lost when playing a given condition.
Fish Reel	0	20	6.75	3.54	Count of how many times participants managed to reel a fish closer to them in a condition.
Fish Unreel	0	14	6.54	3.31	Count of how many times the fishing rod unreeled (the fish trying to escape) in a condition.
PAM rate	0	0.3	0.18	0.13	Normalized count of trials in which participants received help in a condition.
Condition	-	-	-	-	Participants played four conditions: Normal (no PAM), augmented success, input override, and mitigated failure.

**Table 3:** Participant demographics, individual scores per condition (Likert scales of perceived control and frustration), MI conversion rate (% of MI events, which resulted in positive outcomes), and positive feedback (% of trials, which delivered positive feedback). Gray denotes high frustration, low perceived control, low MI conversion rate, or low positive feedback.

Variable	1	3	4	5	6	7	8	9	10	11	12	13	14	15	16	17	18	19
Gender	F	M	M	M	M	F	M	M	F	F	F	F	M	M	M	M	M	F
Age	27	29	60	27	22	23	24	24	23	22	33	24	22	24	21	28	26	25
Perceived Performance	0.85	0.95	0.35	NA	0.7	0.75	0.2	0.75	0.8	0.075	0.6	0.5	0.15	0.35	0.6	0.35	0.45	0.5
BCI Experience	Yes	Yes	No	No	Yes	Yes	Yes	Yes	Yes	Yes	No	No	Yes	No	No	No	Yes	Yes
Perc. Control	0.67	0.75	0.21	0.37	0.63	0.58	0.29	0.54	0.46	0.04	0.29	0.71	0.11	0.54	0.75	0.42	0.38	0.54
Frustration	0.33	0.13	1.00	0.38	0.54	0.42	0.50	0.58	0.42	1.00	0.54	0.25	0.67	0.50	0.29	0.83	0.54	0.21
MI Conv. Rate	92%	85%	21%	61%	32%	80%	32%	75%	36%	11%	71%	80%	27%	52%	34%	45%	78%	55%
Pos. Feedback	78%	74%	28%	57%	35%	70%	35%	68%	40%	18%	66%	70%	32%	50%	40%	49%	65%	52%
Aug. Success																		
Perc. Control	0.67	0.83	0.33	0.33	0.50	0.33	0.33	0.17	0.33	0.00	0.17	0.50	0.33	0.67	1.00	0.67	0.17	0.67
Frustration	0.33	0.17	1.00	0.17	0.67	0.50	0.67	0.83	0.33	1.00	0.50	0.17	0.67	0.33	0.17	0.67	0.67	0.17
MI Conv. Rate	95%	80%	15%	60%	15%	90%	50%	30%	35%	15%	85%	75%	35%	65%	45%	45%	85%	55%
Pos. Feedback	65%	60%	15%	50%	15%	55%	35%	20%	25%	15%	70%	50%	30%	50%	45%	45%	60%	45%
Fish Caught	0	6	1	4	1	5	1	0	2	1	8	4	2	6	5	6	5	5
Fish Lost	0	0	5	1	5	2	4	5	4	5	0	2	3	2	3	2	2	2
Input Override																		
Perc. Control	0.50	0.50	0.17	0.50	0.67	0.67	0.33	0.50	0.50	0.00	0.33	0.67	0.00	0.67	0.83	0.33	0.33	0.33
Frustration	0.50	0.17	1.00	0.50	0.67	0.50	0.33	0.50	0.17	1.00	0.67	0.83	0.50	0.50	0.33	0.83	0.50	0.50
MI Conv. Rate	100%	95%	5%	55%	35%	95%	30%	95%	15%	5%	50%	90%	30%	65%	40%	30%	65%	45%
Pos. Feedback	100%	95%	35%	70%	50%	100%	55%	95%	40%	35%	60%	95%	50%	75%	65%	60%	70%	60%
Fish Caught	0	8	2	6	3	7	5	8	2	2	4	7	3	7	4	4	5	4
Fish Lost	0	0	3	0	2	0	2	0	3	4	1	0	2	0	1	1	0	2
Mit. Failure																		
Perc. Control	0.50	0.67	0.00	0.33	0.67	0.33	0.33	0.67	0.33	0.17	0.00	0.67		0.50	0.67	0.17	0.33	0.67
Frustration	0.33	0.17	1.00	0.33	0.33	0.50	0.33	0.67	0.50	1.00	0.83	0.00		0.50	0.33	1.00	0.50	0.00
MI Conv. Rate	90%	80%	10%	70%	30%	50%	25%	75%	30%	25%	80%	75%		55%	15%	40%	85%	60%
Pos. Feedback	60%	55%	5%	50%	25%	40%	25%	55%	30%	20%	65%	55%		50%	15%	25%	55%	45%
Fish Caught	0	4	0	4	1	2	1	5	1	1	5	4		3	1	1	4	3
Fish Lost	0	0	4	1	2	1	3	0	2	3	0	0		1	3	2	0	1
Ref. Condition																		
Perc. Control	1.00	1.00	0.33	0.33	0.67	1.00	0.17	0.83	0.67	0.00	0.67	1.00	0.00	0.33	0.50	0.50	0.67	0.50
Frustration	0.17	0.00	1.00	0.50	0.50	0.17	0.67	0.33	0.67	1.00	0.17	0.00	0.83	0.67	0.33	0.83	0.50	0.17
MI Conv. Rate	85%	85%	55%	60%	50%	85%	25%	100%	65%	0%	70%	80%	15%	25%	35%	65%	75%	60%
Pos. Feedback	85%	85%	55%	60%	50%	85%	25%	100%	65%	0%	70%	80%	15%	25%	35%	65%	75%	60%
Fish Caught	0	6	3	5	4	8	1	8	4	0	6	6	1	2	2	5	7	4
Fish Lost	0	0	2	1	2	0	4	0	0	6	0	0	5	4	3	0	0	2

**Table 4:** (Top) Results of significant likelihood ratio tests predicting perceived control, with the AIC (Akaike information criterion), ML (maximum likelihood), LR (likelihood ratio), and $\chi^{2}$ (significance). (Bottom) fixed effect estimates for predicting perceived control in the best model “Fish Lost + PAM Rate”.

Predicted	Fixed Effect	AIC	ML	LR	$\chi^{2}$
Perceived Control	Fish Lost + PAM Rate	215.82	−98.91	15.61	<0.001
	Fish Lost + Condition	219.11	−98.55	16.32	0.001
	Fish Lost + Fish Caught	226.70	−104.35	4.72	0.030
	Fish Lost	229.43	−106.71	24.05	<0.001
	Fish Caught	232.12	−108.06	21.36	<0.001
	Pos. Feedback	233.27	−108.63	20.21	<0.001
	MI Conv. Rate	237.67	−110.83	15.81	<0.001
	Fish Reel	242.10	−113.05	11.38	0.001
	Fish Unreel	245.62	−114.81	7.86	0.005
**Predicted**	**Fixed Effect**	**Estimate**	**Std. Error**	**z Value**	***p***
Perceived Control	PAM Rate	−7.86	2.14	−3.68	<0.001
	Fish Lost	−1.39	0.27	−5.11	<0.001

**Table 5:** (Top) Results of significant likelihood ratio tests predicting frustration. (Bottom) fixed effect estimates for predicting frustration in the best model “Fish Lost”.

Predicted	Fixed Effect	AIC	ML	LR	$\chi^{2}$
Frustration	Fish Lost	239.63	−111.82	8.81	0.003
	Fish Caught	240.46	−112.23	7.99	0.005
	MI Conv. Rate	242.49	−113.25	5.95	0.015
	Pos. Feedback	244.20	−114.10	4.24	0.039
**Predicted**	**Fixed Effect**	**Estimate**	**Std. Error**	**z Value**	***p***
Frustration	Fish Lost	0.62	0.21	2.96	0.003

## Data Availability

The data that support the findings of this study are available upon reasonable request from the corresponding author.
